# Case of Supposed Retention and Development of the Placenta after Decomposition and Discharge of Fœtus

**Published:** 1852-08

**Authors:** S. Mitchell

**Affiliations:** Cameron Mills, Steuben Co., N. Y.


ART. V.— *Case of supposed Retention and Development of the Placenta after Decomposition and Discharge of Fœtus.* By S. MITCHELL, M. D., Cameron Mills, Steuben Co., N. Y.

Was called, April 9, 1852, to see Mrs. B——, aged twenty-five, nervous lymphatic temperament, full habit, good constitution, having enjoyed uniformly good health previous to the present difficulty, and borne two children.

She informed me that she became " enciente," about the last of July, 1851, and experienced the usual symptoms attending the early periods of pregnancy, such as suppression of the menses, nausea, vomiting, &c. The abdomen, she thinks, increased in size, about the same as it had done in her previous pregnancies. Sometime in November, as near as she could remember about the middle, she had a fall, which was followed by very great soreness across the abdomen and loins; but was attended with no hæmorrhage or pains denoting uterine contractions. She had felt no motion previous to the accident which must have occurred between the third and fourth months.

The soreness gradually subsided so that she was enabled in the course of two or three weeks to atttend to her usual household duties.

The uterus slowly enlarged after this, and rose above the pelvis near the umbilicus, but she never felt any motion. Some three weeks previous to my seeing the patient, she commenced having a dark offensive sanguineous discharge from the uterus. This continued unabated up to the time the case first came under my observation. By a vaginal examination I found the os firmly closed, not even admitting the tip of the finger, and of a firm and healthy feeling except a slight abrasion, or ulcer, upon the anterior lip. As before described, the uterus reached to near the umbilicus, was flaccid, and quite tender upon pressure, and yet she had no febrile action, the patient only complaining of a constant feeling of lassitude and debility; the countetenance rather anemic, still able to be about house.

*Diagnosis.* If I placed any reliance upon her statement that she became pregnant the last of July, and notwithstanding the accident that befel her, having had no hæmorrhage, or pains denoting uterine contractions by which the ovum could have been expelled, I must of necessity come to the conclusion that it still remained in the uterus, and that the injury was sufficient to suspend its further development, and yet not sufficient to cause its expulsion, consequently it had been retained, harmlessly enclosed within the membranes, until the period when the discharge commenced, when, undoubtedly, the membranes were ruptured, and a decomposition and gradual discharge of the embryo commenced, giving rise to the dark and offensive evacuation before mentioned.

*Treatment.* After deciding that the uterus contained a fœtus in a putrid, decomposing state, which it was making no efforts, by natural contractions, to rid itself of, the question arose in my mind which would be attended with least danger to my patient, to seek by artificial means to dilate the os uteri, and then excite uterine contractions by the use of ergot, or wait the action of nature — "*the vis medicatrix naturæ*" — closely watching the case to guard against every unfavorable indication. I was induced to pursue the latter course by observing how little the system was suffering from the protracted sanguineous discharge.

The ulcer upon the os I touched with nit. silver, and directed the vagina to be injected with chlorine water several times per day, to correct the fetor; volatile liniment to be freely rubbed over the region of the uterus, and advised rest in the horizontal position, and to *wait.*

I saw the patient from time to time; the sanguineous discharge continuing unabated, but becoming much lighter colored and less offensive. The uterus slowly diminishing in size and becoming firmer and less tender to the touch; the patient complaining more and more of debility, countenance becoming more and more anemic, pulse more frequent and smaller, extremities inclined to be cold, and appetite much impaired, I had been for several days thinking of the propriety of abandoning this "*expectant*" plan of treatment and resorting to some more active measures to procure the evacuation of the uterine contents, when I was summoned in great haste April 29, 1852. I found the patient with strong uterine contractions occurring at frequent intervals, which expelled in the course of two hours what appeared to be a fleshy mass of a pyriform shape, in fact being a perfect cast of the uterine cavity, some five inches in length, and three inches its greatest diameter.

The mass I found to be hollow, its walls being made up of a placenta-like structure which was from one to one and a-half inches in thickness at the fundus, and gradually becoming thinner toward the apex, which was membranous. The membranous portion was ruptured, through which the discharge had evidently escaped. From the fundus there was a pyramidal fleshy mass suspended by its base, which nearly filled the cavity. It was soft and friable between the fingers, and of a nearly black color, denoting that decomposition was taking place. It had no appearance of an organized fœtus except its being covered by a smooth membrane which was also reflected over the interior of the cavity. This undoubtedly was once the fœtus, but its further development being suspended by the injury, it degenerated into this anomalous structure. After the evacuation of the uterus the patient became speedily convalescent.

The case is of interest to me by its proving that the placenta may continue its growth and development up to the full period of utero-gestation, even when the fœtus, which it is intended to nourish, is destroyed. The reader will observe that the time of the expulsion of the mass was just nine months after the time fixed upon by the patient as the period of impregnation.

